# Urinary incontinence, mental health and loneliness among community-dwelling older adults in Ireland

**DOI:** 10.1186/s12894-017-0214-6

**Published:** 2017-04-08

**Authors:** Andrew Stickley, Ziggi Ivan Santini, Ai Koyanagi

**Affiliations:** 1 The Stockholm Center for Health and Social Change (SCOHOST), Södertörn University, Huddinge, 141 89 Sweden; 2 The Danish National Institute of Public Health, University of Southern Denmark, Oester Farimagsgade 5A, 1353 Copenhagen, Denmark; 3 Parc Sanitari Sant Joan de Déu, Universitat de Barcelona, Fundació Sant Joan de Déu/CIBERSAM, Barcelona, Spain

**Keywords:** Urinary incontinence, Lonely, Anxiety, Depression

## Abstract

**Background:**

Urinary incontinence (UI) is associated with worse health among older adults. Little is known however, about its relation with loneliness or the role of mental health in this association. This study examined these factors among older adults in Ireland.

**Methods:**

Data were analyzed from 6903 community-dwelling adults aged ≥ 50 collected in the first wave of The Irish Longitudinal Study on Ageing (TILDA) in 2009–11. Information was obtained on the self-reported occurrence (yes/no) and severity (frequency/activity limitations) of UI in the past 12 months. Loneliness was measured using the UCLA Loneliness Scale short form. Information was also obtained on depression (CES-D), anxiety (HADS-A) and other sociodemographic variables. Logistic regression analysis was used to examine the association between variables.

**Results:**

In a model adjusted for all potential confounders except mental disorders, compared to no UI, any UI was associated with significantly higher odds for loneliness (odds ratio: 1.51). When depression was included in the analysis, the association was attenuated and became non-significant while the inclusion of anxiety had a much smaller effect. Similarly, although frequency of UI and activity limitations due to UI were both significantly associated with loneliness prior to adjustment for mental disorders, neither association remained significant after adjustment for both depression and anxiety.

**Conclusion:**

UI is associated with higher odds for loneliness among older community-dwelling adults but this association is largely explained by comorbid mental health problems, in particular, depression.

## Background

Urinary incontinence (UI), which is defined as the involuntary leakage of urine [1] is highly prevalent in the general population and can severely affect many aspects of daily life [2, 3]. Although this condition can exist in adults of all ages [3], a large body of research has shown that the prevalence of UI increases with age [4, 5] and that the elderly are especially vulnerable to this condition [6] particularly in a severe form [7, 8]. While previously reported prevalence figures vary due to the different operational definitions of UI employed (type, severity etc.), an earlier review article presented figures which showed that the prevalence of UI ranges between 9 and 59% in those aged 50 and above [9].

Studies have indicated that UI can have a significant negative effect on the lives of older people [10]. For example, it has been associated with troublesome symptoms such as aches, pain, weakness, and shortness of breath [11], as well as with an increased risk for outcomes such as falls and fractures [12, 13]. The avoidance of physical activity in an attempt to manage/control the condition may also have an effect on overall health by increasing the risk of conditions such as hypertension [14, 15]. In addition, UI is also associated with poorer mental health among older persons including anxiety disorders [16] and depression [16, 17].

Despite the large number of studies on UI and its associated adverse health outcomes, one condition which has been little studied to date in relation to UI is loneliness. This is an important research gap given that: (a) incontinent individuals can experience feelings of frustration, embarrassment and shame [18, 19] as a result of their condition and will sometimes reduce/avoid social contacts and activities in order to control UI and its effects [18], which may lead to increased social isolation and feelings of loneliness; and (b) loneliness has itself been linked to an increased risk for morbidity and mortality among older persons [20, 21]. To the best of our knowledge, to date, there have been only three studies which have investigated this association. Specifically, two recent studies have shown that older adults (≥57 years old) with UI in Canada and the United States have an increased risk of feeling lonely compared to those who are continent [22] or who have no/less severe UI symptoms (no/weekly/monthly/yearly vs. daily) [23], respectively. An earlier study from the United States also found similar results where UI and UI severity (measured by the quantity of urine loss) were both associated with loneliness among middle-aged and older adults (≥ 40 years old) [24].

Although these studies have advanced understanding of the psychological consequences of UI, there are aspects of the association between UI and loneliness among older adults that are yet to be elucidated. In particular, there has been an absence of research on the role of common mental disorders (CMDs) in this association. This is an important gap in the research as not only are anxiety and depression linked to UI in older persons [16, 17], but other research has highlighted their close link with loneliness in older adults [25–27] and that in middle-aged and older adults, depressive symptoms and loneliness may be reciprocally related [28]. Therefore, there is a need to assess the extent to which CMDs explain the association between UI and loneliness. In addition, until now, there has been no research on whether the specific consequences of UI, such as activity limitations, are important for loneliness in older adults.

Thus, using data from a nationally representative sample of community-dwelling older adults (aged 50 and above) in Ireland, the current study had three aims: (1) to determine if UI is associated with an increased risk of feeling lonely; (2) to examine if the severity of UI, as measured by the frequency of urine loss and activity limitations, is associated with loneliness; and (3) to assess the role of CMDs in the association between UI, UI severity and loneliness.

## Methods

### Study design and sample

The data used in this study came from the first wave of The Irish Longitudinal Study on Ageing (TILDA) which was conducted by Trinity College Dublin between October 2009 and February 2011. Details of the survey and its sampling procedure have been published previously [29, 30]. In brief, TILDA was a nationally representative survey of community-based adults aged 50 and above living in Ireland. The target sample included every household resident meeting this age criterion. Clustered random sampling was used to obtain a nationally representative sample. Individuals who were institutionalized and those who had doctor-diagnosed dementia were excluded. If severe cognitive impairment (judged at the interviewer’s discretion) prevented individuals from providing written informed consent to participate in the survey, they were also excluded [31]. The data was collected by trained interviewers using computer-assisted personal interviewing (CAPI), and with the use of self-completion questionnaires (SCQs). All individuals that underwent a CAPI interview were also asked to complete the SCQ. The overall response rate was 62%, while 84% of those who participated in the survey returned the SCQ [29, 30].

In total, 8504 people aged ≥50 years (*n* = 8175) and their spouses or partners younger than 50 years (*n* = 329) comprised the survey sample. In the current study, the analysis was restricted to participants aged 50 years and above and those who completed the SCQ. These conditions were necessary as information on certain variables (e.g., loneliness, anxiety etc.) was obtained from the SCQ. Following these restrictions, the analytic sample comprised 6903 individuals. The Faculty of Health Sciences Ethics Committee of Trinity College Dublin provided ethical approval for TILDA, with written informed consent being obtained from all participants.

### Measures

#### Loneliness (Dependent variable)

The short form of the University of California, Los Angeles (UCLA) Loneliness Scale was used to assess feelings of loneliness [32, 33]. The short form UCLA Loneliness Scale, which assesses subjective feelings of social isolation, is a commonly used measure in loneliness research. The dominant factor underlying the UCLA Loneliness scale is ‘perceived social isolation’ [34, 35]. The UCLA three-item scale is comprised of three negatively-worded questions relating to feelings of isolation, feeling left out and companionship. The three response options are coded as 1 (hardly ever), 2 (some of the time), and 3 (often). Scores are summed to create a total score that runs from 3 to 9, with higher scores indicating a greater degree of loneliness (Cronbach’s alpha = 0.81). Previous research has indicated that this scale has an acceptable degree of reliability and has both concurrent and discriminant validity [33]. As the distribution of the loneliness variable was right-skewed, in this study we used a dichotomous loneliness variable for the regression analyses. Specifically, in accordance with a recent study, a score of 4–9 was categorized as feeling lonely while a score of 3 (i.e., replying ‘hardly ever’ to all of the questions) was classified as not feeling lonely [22].

#### Urinary incontinence (UI) (Independent variable)

Any UI was assessed by the question ‘During the last 12 months, have you lost any amount of urine beyond your control?’ with the answer options ‘yes’ or ‘no’. For those who responded affirmatively to this question, follow-up questions on the frequency of UI and limitations in activity due to UI were asked. Frequency was assessed by the question ‘Did this happen more than once during a 1 month period?’ and activity limitations were examined by the question ‘Do you ever limit your activities, for example, what you do or where you go, because of UI?’ Both of these questions had ‘yes’ or ‘no’ as answer options.

#### Depression

Depressive symptoms were measured with the 20-item Center for Epidemiologic Studies Depression (CES-D) scale [36], which assesses symptoms experienced in the preceding week. Its 20 items are scored on a scale from 0 (rarely or none of the time, less than one day in the week) to 3 (most or all of the time, five to seven days in the week). In order to avoid an overlap with the outcome (loneliness), and following the lead of an earlier study [37], we excluded the item on loneliness (‘I felt lonely’) that is included in the CES-D scale. Thus, scores from the remaining 19 items were summed to create a scale with values ranging from 0 to 57 where higher scores signified more depressive symptoms (Cronbach’s alpha = 0.87). Previous studies have highlighted the validity of the CES-D scale as a measure of depression in community-dwelling older adults [38, 39].

#### Anxiety

The Hospital Anxiety and Depression Scale (HADS-A) [40] was used to assess anxiety symptoms. This scale measures the presence of anxiety symptoms without reference to a specific time frame. The scale consists of seven items rated on a four-point scale from 0 (not at all) to 3 (very often indeed), five of which are reverse coded. The scores from the individual items were summed to create a total score that ranged from 0 to 21, with higher scores indicating more anxiety (Cronbach’s alpha = 0.65). Previous research has indicated that the HADS is a reliable measure in both younger and older persons [41].

#### Control variables

##### Social network index

The Berkman-Syme Social Network Index (SNI) was used to assess social networks. The SNI is a validated self-report questionnaire [42] that assesses the degree to which a person is socially integrated. Information is elicited on marital/partnership status (married/with partner versus not), sociability (number of children, close relatives, and close friends and the frequency of contact with them), and church group or community organization membership. A composite score is calculated that ranges from 0 to 4. In this study, we used what is regarded as the standard categorization [i.e., 0–1 (most isolated), 2 (moderately isolated), 3 (moderately integrated), and 4 (most integrated)] [42]. Further information on the psychometric properties of the SNI and evidence relating to its predictive validity has been provided elsewhere [43].

#### Chronic medical conditions

To assess chronic health conditions, participants were presented with a list of 17 medical conditions and asked, “has a doctor ever told you that you have any of the conditions on this card?” These conditions were: high blood pressure or hypertension; angina; heart attack (including myocardial or coronary thrombosis); congestive heart failure; diabetes or high blood sugar; stroke (cerebral vascular disease); ministroke or transient ischemic attack; high cholesterol; heart murmur; abnormal heart rhythm; any other heart trouble; chronic lung disease such as chronic bronchitis or emphysema; asthma; arthritis (including osteoarthritis, or rheumatism); osteoporosis; cancer or a malignant tumor (including leukemia or lymphoma but excluding minor skin cancers); cirrhosis or serious liver damage. The total number of chronic medical conditions was calculated and divided into three categories: 0 (none), 1, or ≥2.

#### Activities of daily living (ADL) disability

To assess ADL disability participants were asked to indicate whether they had difficulty performing six activities (dressing, walking, bathing, eating, getting in or out of bed, and using the toilet) [44]. Participants having difficulty with one or more ADLs were categorized as having an ADL disability.

#### Sociodemographic variables

Sociodemographic characteristics included age (50–59, 60–69, 70–79, and ≥80 years), sex, education, and wealth. Education was divided into three categories: primary (some primary/not complete; primary or equivalent); secondary (intermediate/junior/group certificate or equivalent; leaving certificate or equivalent); and tertiary (diploma/certificate; primary degree; postgraduate/higher degree). As more than 50% of the income values were missing, a proxy measure (financial strain) was used to assess wealth. Participants were thus asked to respond to the statement that a ‘shortage of money stops me from doing the things I want to do’ using one of the answer options, ‘never’, ‘rarely’, ‘sometimes’, and ‘often’.

### Statistical analysis

Stata version 14.1 (Stata Corp LP, College Station, Texas) was used to perform the analysis. In the first stage, descriptive statistics are presented of the study sample. The difference in sample characteristics by the presence of UI was tested by using Chi-square and Student’s t-tests for categorical and continuous variables, respectively. Logistic regression analysis was then used to firstly assess the association between any UI (independent variable) and loneliness (dependent variable) based on the question ‘During the last 12 months, have you lost any amount of urine beyond your control?’. A hierarchical analysis was conducted by including different variables sequentially in different models to assess how these variables influenced the association between UI and loneliness. Six different models were thus constructed: Model 1: unadjusted; Model 2: adjusted for age, sex, education, financial strain, number of chronic conditions, and ADL disability; Model 3: adjusted for the variables in Model 2 and the SNI; Model 4: adjusted for the variables in Model 3 and depression; Model 5: adjusted for the variables in Model 3 and anxiety; Model 6: adjusted for the variables in Model 3, depression, and anxiety. The selection of the variables used for adjustment was based on past literature.

To assess the association between UI severity and loneliness, we repeated the analytic method described above but replaced the any UI variable with a three-category UI variable which incorporates the frequency of urinary inconsistence [UI (-); UI (+) once a month or less; UI (+) more than once a month], or activity limitations due to UI [UI (-); UI (+) but no activity limitations; UI (+) with activity limitations]. This analysis used ‘no UI’ as the reference category. Finally, we also performed this analysis while restricting it to those with UI to assess whether the frequency of UI or activity limitations due to UI confers an increased risk for loneliness among those with UI. All variables included in the models were categorical variables apart from depression and anxiety which were continuous variables. The dataset also included sampling weights that were created based on the age, sex and educational attainment values in the Quarterly National Household Survey 2010. In order to obtain nationally representative estimates, the sample weighting and the complex study design, including within household clustering, was taken into account in all analyses. Results are expressed as odds ratios (OR) and 95% confidence intervals (95% CIs). A *p*-value <0.05 was considered to be statistically significant.

## Results

**Fig. 1:**
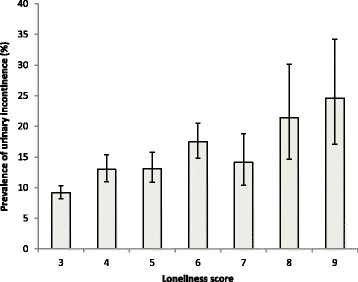
Prevalence of urinary incontinence by loneliness score. Bars denote 95% confidence intervals. Estimates are based on the weighted sample. Urinary incontinence was assessed by the question ‘During the last 12 months, have you lost any amount of urine beyond your control?’ with answer options ‘yes’ or ‘no’. The loneliness score was based on the short form UCLA loneliness scale with higher scores indicating greater levels of loneliness

The mean age (standard deviation) of the sample was 63.6 (9.2) years and 52.1% were women. Overall, the prevalence of any UI was 12.4% (95% CI = 11.5–13.4%). Among those with UI, it occurred more than once a month in 76.6%, and 26.4% had activity limitations due to UI. The sample characteristics are shown in Table 1. Older age, female sex, lower education, financial strain, a higher number of chronic conditions, ADL disability, less social network integration, depression, and anxiety were all significantly associated with UI. The prevalence of any UI by the level of loneliness is illustrated in Fig. 1. Greater loneliness was associated with a higher prevalence of UI with the prevalence of UI ranging from 9.2% (lowest level of loneliness) to 24.6% (highest level of loneliness). The results of the logistic regression analysis assessing the association between any UI and loneliness are shown in Table 2. In the unadjusted model, the OR (95% CI) was 1.74 (1.49-2.05) (Model 1). This was attenuated when the model was adjusted for sociodemographic factors, chronic conditions, and ADL disability but remained statistically significant (Model 2). Further adjustment for the SNI had little effect on the association (Model 3). The OR became non-significant when depression was included in the model (Model 4) but not when anxiety was included (Model 5). In the final model adjusting for all potential confounders the OR (95% CI) was 1.14 (0.94–1.37) (Model 6).

**Table 1:** Sample characteristics (overall and by urinary incontinence)

			Urinary incontinence		
Characteristic	Categories	Overall	No	Yes	*P*-value^a^
Age (years)	50–59	40.5	41.9	30.3	<0.001
	60–69	30.7	30.9	29.8	
	70–79	20.0	19.2	25.1	
	≥80	8.8	7.9	14.9	
Sex	Male	47.9	51.4	23.8	<0.001
	Female	52.1	48.6	76.2	
Education	Primary	38.1	37.2	44.0	<0.001
	Secondary	43.3	44.1	38.3	
	Tertiary	18.6	18.8	17.6	
Financial strain	Never	23.1	23.4	20.7	<0.001
	Rarely	21.6	21.7	21.5	
	Sometimes	36.4	36.8	33.3	
	Often	18.9	18.1	24.6	
Number of	None	23.4	25.2	11.5	<0.001
chronic conditions	One	28.1	28.8	22.9	
	Two or more	48.5	46.0	65.6	
ADL disability	No	90.9	92.7	78.7	<0.001
	Yes	9.1	7.3	21.3	
Social Network Index	Most isolated	7.5	7.0	10.5	0.011
	Moderately isolated	28.8	28.9	27.4	
	Moderately integrated	41.0	41.1	40.3	
	Most integrated	22.7	22.9	21.9	
Depression	Mean (SD)	5.7 (6.8)	5.2 (6.4)	9.1 (8.6)	<0.001
Anxiety	Mean (SD)	5.5 (3.7)	5.3 (3.6)	6.7 (4.1)	<0.001

The data are column percentages unless otherwise stated

Estimates are based on weighted sample

*Abbreviation: ADL* Activities of daily living, *SD* Standard deviation

^a^The difference in sample characteristics by urinary incontinence was tested by Chi-square tests and Student’s *t*-tests for categorical and continuous variables, respectively

**Table 2:** Association between urinary incontinence (independent variable) and loneliness (dependent variable) estimated by logistic regression

Characteristic	Categories	Model 1	Model 2	Model 3	Model 4	Model 5	Model 6
Urinary incontinence	No	Ref	Ref	Ref	Ref	Ref	Ref
	Yes	1.74***	1.50***	1.51***	1.20	1.27*	1.14
		[1.49,2.05]	[1.27,1.78]	[1.27,1.80]	[1.00,1.43]	[1.06,1.53]	[0.94,1.37]
Age (years)	50–59		Ref	Ref	Ref	Ref	Ref
	60–69		0.95	1.03	1.13	1.22**	1.27**
			[0.83,1.08]	[0.90,1.17]	[0.99,1.30]	[1.06,1.41]	[1.10,1.46]
	70–79		1.19*	1.30**	1.43***	1.74***	1.76***
			[1.01,1.40]	[1.10,1.53]	[1.20,1.70]	[1.46,2.07]	[1.47,2.10]
	≥80		1.46**	1.36*	1.53**	2.05***	2.06***
			[1.13,1.88]	[1.05,1.77]	[1.17,1.99]	[1.56,2.70]	[1.56,2.72]
Sex	Male		Ref	Ref	Ref	Ref	Ref
	Female		1.12*	1.10	0.98	0.92	0.87*
			[1.01,1.24]	[1.00,1.22]	[0.88,1.08]	[0.83,1.03]	[0.78,0.98]
Education	Primary		Ref	Ref	Ref	Ref	Ref
	Secondary		0.95	1.03	1.07	1.07	1.08
			[0.82,1.09]	[0.89,1.19]	[0.92,1.24]	[0.92,1.25]	[0.93,1.27]
	Tertiary		0.92	1.04	1.11	1.13	1.15
			[0.80,1.07]	[0.90,1.21]	[0.95,1.29]	[0.96,1.32]	[0.98,1.35]
Financial strain	Never		Ref	Ref	Ref	Ref	Ref
	Rarely		1.38***	1.41***	1.44***	1.21*	1.24*
			[1.18,1.62]	[1.20,1.66]	[1.21,1.70]	[1.02,1.43]	[1.04,1.48]
	Sometimes		1.87***	1.88***	1.80***	1.53***	1.54***
			[1.63,2.16]	[1.63,2.17]	[1.55,2.09]	[1.32,1.79]	[1.32,1.81]
	Often		3.66***	3.35***	2.66***	2.14***	1.99***
			[3.03,4.42]	[2.77,4.06]	[2.17,3.26]	[1.75,2.61]	[1.61,2.45]
Number of	None		Ref	Ref	Ref	Ref	Ref
Chronic conditions	One		1.01	1.04	1.02	1.03	1.02
			[0.86,1.18]	[0.89,1.22]	[0.87,1.20]	[0.87,1.22]	[0.87,1.21]
	Two or more		1.24**	1.25**	1.15	1.12	1.08
			[1.07,1.43]	[1.07,1.44]	[0.99,1.34]	[0.96,1.31]	[0.93,1.27]
ADL disability	No		Ref	Ref	Ref	Ref	Ref
	Yes		1.14	1.06	0.74**	0.94	0.76*
			[0.93,1.39]	[0.87,1.31]	[0.59,0.92]	[0.75,1.18]	[0.60,0.97]
Social Network	Mostly isolated			Ref	Ref	Ref	Ref
Index	Moderately isolated			0.59***	0.63**	0.60***	0.63**
				[0.45,0.77]	[0.47,0.83]	[0.45,0.80]	[0.46,0.84]
	Moderately integrated			0.40***	0.46***	0.40***	0.43***
				[0.31,0.52]	[0.35,0.60]	[0.30,0.52]	[0.32,0.57]
	Most integrated			0.26***	0.30***	0.25***	0.28***
				[0.20,0.34]	[0.23,0.40]	[0.19,0.33]	[0.21,0.37]
Depression	(per one-unit increase)				1.10***		1.06***
					[1.09,1.11]		[1.05,1.07]
Anxiety	(per one-unit increase)					1.24***	1.20***
						[1.21,1.26]	[1.17,1.22]

Data are odds ratio [95% confidence interval]

Models are adjusted for all the variables in the respective columns

*Abbreviation: Ref* Reference category, *ADL* Activities of daily living

*p < 0.05, **p < 0.01, ***p < 0.001

When the frequency of UI or activity limitations due to UI were taken into account, compared to no UI, having activity limitations due to UI was associated with particularly high odds for loneliness even in models adjusted for either depression or anxiety (Model 4 and 5) although the OR was no longer significant when depression and anxiety were included simultaneously in the model (Model 6). Frequency of UI was not as strongly associated with loneliness as activity limitations due to UI and became non-significant in the models where depression and anxiety were included (Table 3). Finally, in the analysis restricted to those with UI, a  higher frequency of UI was not associated with elevated odds for loneliness, but activity limitations due to UI were associated with significantly higher odds for loneliness in all models except those which adjusted for depression (Table 4).

**Table 3:** Association between frequency of urinary incontinence or activity limitations due to urinary incontinence (independent variables) and loneliness (dependent variable) estimated by logistic regression with no urinary incontinence as the reference category

Characteristic	Categories	Model 1	Model 2	Model 3	Model 4	Model 5	Model 6
Frequency of	No urinary incontinence	Ref	Ref	Ref	Ref	Ref	Ref
Urinary incontinence	Once a month or less	1.62**	1.50*	1.53*	1.33	1.25	1.21
		[1.20,2.20]	[1.08,2.07]	[1.09,2.14]	[0.94,1.90]	[0.88,1.78]	[0.85,1.74]
	More than once a month	1.79***	1.51***	1.52***	1.16	1.29*	1.11
		[1.49,2.15]	[1.24,1.83]	[1.25,1.84]	[0.94,1.42]	[1.04,1.59]	[0.90,1.38]
Activity limitations	No urinary incontinence	Ref	Ref	Ref	Ref	Ref	Ref
Due to urinary	No activity limitations	1.53***	1.36**	1.37**	1.13	1.16	1.06
Incontinence		[1.28,1.84]	[1.12,1.64]	[1.12,1.67]	[0.92,1.39]	[0.94,1.43]	[0.86,1.32]
	Activity limitations	2.60***	2.07***	2.08***	1.46*	1.71**	1.41
		[1.91,3.55]	[1.51,2.84]	[1.50,2.88]	[1.03,2.05]	[1.20,2.45]	[0.98,2.04]

Data are odds ratio [95% confidence interval]

Model 1: Unadjusted

Model 2: Adjusted for age, sex, education, financial strain, number of chronic conditions, and ADL disability

Model 3: Adjusted for variables in Model 2 and the Social Network Index

Model 4: Adjusted for variables in Model 3 and depression

Model 5: Adjusted for variables in Model 3 and anxiety

Model 6: Adjusted for variables in Model 3, depression, and anxiety

*Abbreviation: Ref* Reference category

*p < 0.05, **p < 0.01, ***p < 0.001

**Table 4:** Association between frequency of urinary incontinence or activity limitations due to urinary incontinence (independent variables) and loneliness (dependent variable) restricted to individuals with urinary incontinence estimated by logistic regression

Characteristic	Categories	Model 1	Model 2	Model 3	Model 4	Model 5	Model 6
Frequency of	Once a month or less	Ref	Ref	Ref	Ref	Ref	Ref
Urinary incontinence	More than once a month	1.10	0.99	0.99	0.87	1.04	0.91
		[0.78,1.56]	[0.69,1.43]	[0.68,1.44]	[0.59,1.29]	[0.71,1.54]	[0.61,1.36]
Activity limitations	No activity limitations	Ref	Ref	Ref	Ref	Ref	Ref
Due to urinary	Activity limitations	1.70**	1.52*	1.54*	1.30	1.51*	1.34
Incontinence		[1.20,2.41]	[1.05,2.20]	[1.06,2.24]	[0.87,1.93]	[1.00,2.28]	[0.88,2.04]

Data are odds ratio [95% confidence interval]

Model 1: Unadjusted

Model 2: Adjusted for age, sex, education, financial strain, number of chronic conditions, and ADL disability

Model 3: Adjusted for variables in Model 2 and the Social Network Index

Model 4: Adjusted for variables in Model 3 and depression

Model 5: Adjusted for variables in Model 3 and anxiety

Model 6: Adjusted for variables in Model 3, depression, and anxiety

*Abbreviation: Ref* Reference category

*p < 0.05, **p < 0.01

## Discussion

Using data from a nationally representative sample of community-dwelling older Irish adults, this study showed that having any UI was associated with an increased risk for loneliness. When depression or anxiety was included in the analysis ORs were attenuated, particularly for depression, which suggests that this association is mainly explained by depression. Worse mental health was also important in the relation between UI severity and loneliness as depression fully attenuated the significant association between an increased frequency of UI and loneliness, while an association between activity limitations and loneliness became non-significant when both depression and anxiety were included in the fully adjusted model. When the analysis was restricted to those with UI, depression alone fully attenuated the significant association that was observed between activity limitations and loneliness.

The finding that UI was associated with loneliness when not adjusting for mental health conditions, accords with the results of earlier studies in Canada and the United States [22–24]. This result seems plausible given that UI has been linked to a range of ‘safety-seeking behaviors’ that are used to manage the condition and its effects, such as avoiding contact with others, intimacy and activities outside the home [45] that might all lead to social isolation among those with UI and give rise to feelings of loneliness. Moreover, the results from the analyses examining UI severity also seem to support this idea as activity limitations were strongly associated with loneliness in the whole sample and when the analysis was restricted to those with UI. Being treated differently by other people because of their condition [18] might also act to isolate those with UI and lead to feelings of loneliness, especially as a recent study from the United States has indicated that older women with daily UI often feel left out and that they lack companionship [23].

When the common mental disorder variables, in particular, depression, were entered into the analysis, however, the association between UI, UI severity and loneliness became non-significant. Together with our finding that those with UI are more likely to experience greater anxiety and depression, this suggests that poorer mental health might be an intervening variable between UI and loneliness. It can only be speculated what underlies the association between depression and loneliness among those with UI, as even though earlier research has indicated that they can both influence each other over time [28], as yet, there has been comparatively little research on the specific mechanisms linking depression to loneliness [27]. It is possible, for example, that certain psychological resources might be important in this context. Specifically, a recent study has reported that a lower sense of mastery significantly contributes to the association between depression and (emotional) loneliness [27] while other research has indicated that UI is associated with a lower sense of mastery [46] and that there is an association between a poor sense of mastery and depression in those with UI [47]. One of the safety-seeking behaviors among those with UI – inquiring frequently if he or she smells [45] – might also be a factor that links depression and loneliness, as a more general connection has been shown to exist between seeking reassurance excessively and both depression and interpersonal rejection [48].

There are several limitations that should be borne in mind when considering this study’s findings. UI data were self-reported in this study. Given the stigma and embarrassment that is associated with UI, it is possible that underreporting may have been an issue [4], although the prevalence estimate obtained fell within the range of those reported in other studies. We also lacked information on the type of UI that was experienced. This might be an important omission as there is some evidence that urge incontinence may affect well-being more than stress incontinence [11] and have a stronger association with worse mental health [49]. There may have also been a problem with one of the instruments used in this study. Specifically, a recent systematic review has questioned the ability of HADS to clearly differentiate between anxiety and depression and indicated that it might be better regarded as a measure of ‘emotional distress’ [50]. In addition, since individuals with cognitive impairment that was severe enough to preclude participation in the survey, and the institutionalized were not included in our study sample, the study results cannot be generalized to this population. Finally, as this study was cross-sectional, it was not possible to determine causality or the temporality of the observed associations.

## Conclusion

This study, which used data from a nationally representative sample of almost 7000 community dwelling adults aged ≥ 50, has shown that UI and UI severity are linked to loneliness but that this association is largely dependent on the presence of comorbid depression. The results of this study and the detrimental (psychological/mental health) outcomes that have been reported in earlier studies, together with the fact that at least one-third of older adults with UI do not seek help [14], suggest that more effort is required to educate older respondents about this condition and its effects, as well as about the wide variety of treatment options that are available for it [51]. For patients, clinician screening for loneliness and then referral to agencies that run social programs (e.g. group meals) that might help alleviate this phenomenon [52] may be one way to improve the quality of life in those individuals with UI. In addition, as poorer mental health is more prevalent among people with UI, and can affect the course and outcome of UI [49, 53], routine mental health screening and close collaboration with mental health professionals may also prove efficacious for patients with UI.

## Abbreviations

95% CI: 95% confidence interval
ADL disability: Activities of daily living disability
CAPI: Computer-assisted personal interviewing
CES-D: Center for Epidemiologic Studies Depression scale
CMDs: Common mental disorders
HADS-A: The Hospital Anxiety and Depression Scale
OR: Odds ratio
SCQ: Self-completion questionnaire
SNI: The Berkman-Syme Social Network Index
TILDA: The Irish Longitudinal Study on Ageing
UCLA Loneliness Scale: University of California, Los Angeles Loneliness Scale
UI: Urinary incontinence
